# Theracurmin Modulates Cardiac Inflammation in Experimental Model of *Trypanosoma cruzi* Infection

**DOI:** 10.3390/tropicalmed8070343

**Published:** 2023-06-28

**Authors:** Vitória Louise, Bianca Alves Almeida Machado, Washington Martins Pontes, Tatiana Prata Menezes, Fernanda Carolina Ribeiro Dias, Luiz Otávio Guimarães Ervilhas, Kelerson Mauro de Castro Pinto, André Talvani

**Affiliations:** 1 Health and Nutrition Post-Graduate Program, Federal University of Ouro Preto, Ouro Preto 35402-145, Minas Gerais, Brazil; vitoria.louise@aluno.ufop.edu.br (V.L.); washington.pontes@aluno.ufop.edu.br (W.M.P.); tatiana.prata@yahoo.com.br (T.P.M.); 2 Medical School, Federal University of Ouro Preto, Ouro Preto 35402-145, Minas Gerais, Brazil; bianca.machado@aluno.ufop.edu.br; 3 Department of Veterinary Medicine, Rural Federal University of Pernambuco, Recife 52171-900, Pernambuco, Brazil; fernandaribeiro.dias@hotmail.com; 4 Department of General Biology, Federal University of Viçosa, Viçosa 36570-000, Minas Gerais, Brazil; luizotavioguimaraes_@hotmail.com; 5 School of Physical Education, Federal University of Ouro Preto, Ouro Preto 35402-145, Mina Gerais, Brazil; kelerson@ufop.edu.br; 6 Infectology and Tropical Medicine Post-Graduate Program, Federal University of Minas Gerais, Belo Horizonte 30130-100, Minas Gerais, Brazil

**Keywords:** Theracurmin, *Trypanosoma cruzi*, inflammation, cardiac tissue, IL-15, CCL2

## Abstract

Theracurmin is a nanoparticle formulation derived from curcumin, a bioactive compound known for its antioxidant and anti-inflammatory properties. *Trypanosoma cruzi*, the etiological agent of Chagas disease, triggers an intense inflammatory response in mammals and also causes severe tissue damage. To evaluate the immunomodulatory and antiparasitic effects of Theracurmin, Swiss mice were experimentally infected with 10^3^ trypomastigote forms of the Colombian strain of *T. cruzi* and submitted to daily therapy with 30 mg/kg of Theracurmin. In addition, daily benznidazole therapy (100 mg/kg) was performed as a positive control. We evaluated the systemic and tissue parasitism, the survival and the body mass rate, the release of inflammatory mediators (TNF, IL-6, IL-15, CCL2 and creatine kinase) and the tissue inflammation at day 30 post-infection. Theracurmin therapy reduced the parasitemia curve without altering the animals’ survival rate, and it protected mice from losing body mass. Theracurmin also reduced CCL2 in cardiac tissue, IL-15 in cardiac and skeletal tissue, and plasma CK. Even without effects on TNF and IL-6 production and tissue amastigote nests, Theracurmin reduced the leukocyte infiltrate in both evaluated tissues, even in the case of more effective results observed to the benznidazole treatment. Our data suggest Theracurmin has an immunomodulatory (CCL2, IL-15, CK and tissue leukocyte infiltration) and a trypanocidal effect (on circulating parasites) during experimental infection triggered by the Colombian strain of *T. cruzi*. Further investigations are necessary to comprehend the Theracurmin role performed in combination with benznidazole or other potential anti-*T. cruzi* chemical compounds.

## 1. Introduction

Curcumin [1,7-bis(4-hydroxy-3-methoxyphenyl) hepta-1,6-diene-3,5-dione)] is a hydrophobic polyphenol, essentially found in *Curcuma longa* L. (Zingiberaceae), a native plant of Southeast Asia which is commonly used for food consumption purposes. This nutraceutical is considered to be one of the main compounds in the plant’s rhizome and it is also characterized by its pleiotropic activities generated from its ability to modulate signaling molecules such as NK-kB, STAT3, cyclooxigenases 1 and 2, prostaglandin-E2, apoptotic proteins and distinct inflammatory mediators [1]. The antioxidant and anti-inflammatory properties investigated in metabolic, cardiovascular, gastrointestinal and tumor diseases [2] have made it a key target of investigation in tropical disease area.

Curcumin has recently been applied in therapeutic strategies against the protozoan *Trypanosoma cruzi* [3,4], the causative agent of human Chagas disease (CD). The *T. cruzi*-triggered pathogenesis is multifactorial and characterized by systemic and progressive muscle tissue inflammation triggered by molecules from the parasites, such as extracellular vesicles, glycosylphosphatidyl inositol (GPI)-anchored glycoconjugates, ectonucleoside triphosphate diphosphohydrolase, mucin-associated surface proteins, GP85/trans-sialidases and others [5,6]. The elimination of blood and tissue parasites is essential to the modulation of the immune response, which can cause muscle cell destruction, apoptosis, local fibrosis and it may also be responsible for the missing of the functional and structural architecture of the infected organ. Currently, the front-line nitroheterocyclic drugs, benznidazole and nifurtimox, used as treatment for CD are related to low efficacy in the chronic phase and to adverse effects and cytotoxicity [7]. New chemical or naturally derived compounds are extremely desirable when aiming at trypanocide and immunoregulatory effects on infected tissues [8,9].

Despite curcumin’s immunomodulatory action, its low absorption capacity and partial water solubility limit its actions as a therapeutic supplement [10]. Therefore, Theracurmin^®^ (Theravalues, Tokyo, Japan) has been proposed as a formulation that amplifies the absorption and bioavailability, those previously described parameters, and it also maintains the therapeutic activities of curcumin in an experimental model [11]. Theracurmin is a smaller particle (0.19 μm) than curcumin (22.75 μm) and reaches the maximum plasma concentration at 1.5 to 3 h, while curcumin powder takes 18.4 to 20.5 h in humans [12]. Therefore, the present study has evaluated the potential protective and trypanocidal effects of Theracurmin in mice infected with the Colombian strain of *T. cruzi* during the initial phase of infection.

## 2. Materials and Methods

Swiss male mice aged 7–9 weeks were either infected or not with 10^3^ blood trypomastigote forms of the Colombian strain of *T. cruzi*, which was isolated from blood culture from an individual in Colombia [13] and classified in discrete typing units (DTU) TcI due to its cardiac tropism, magnificence of the cardiac inflammatory infiltration and of the tissue pro-inflammatory mediators, and resistance to benznidazole and nifurtimox [14,15]. Animals were maintained in autoclavable polypropylene boxes, at the Center of Animal Science of UFOP. Animals were grouped (*n* = 8) into (i) uninfected/untreated, (ii) Theracurmin, (iii) *T. cruzi* and (iv) *T. cruzi* + Theracurmin. They were treated with 30 mg/kg/day [16] of Theracurmin (CurcuminRich^®^, Natural Factors, Coquitlam, BC, CA) by gavage for 30 days, every morning. This compound consists of a nanoparticle colloidal dispersion, made of 10% *w*/*w* curcumin, 2% other curcuminoids such as demethoxycurcumin and bisdemethoxycurcumin, 46% glycerin, 4% ghatti gum and 38% water. The solution was made of Theracurmin powder and distilled water, and the untreated animals received the same vehicle, daily, by gavage. Theracurmin administration (300 μL/animal) or the vehicle started at the day before the infection and the dose was readjusted weekly according to the average body mass gain of the animals. In addition, after confirmation of *T. cruzi* infection, benznidazole (100 mg/kg) was orally administrated as positive control therapy [17].

The count of circulating parasites in the blood of infected animals was performed daily using the parasitemia technique, which consists of reading blood samples (5 μL) through an optical microscope [18]. With a cut of approximately 1 mm, the blood was taken from the tail veins. Mortality data were also collected daily, and body mass was assessed once a week using a digital scale. The euthanasia was performed on day 30 post infection and blood and cardiac and gastrocnemius muscles were collected to biochemical and immunological assays.

Levels of TNF, IL-6, IL-15, IL-10 and CCL2 were detected in the supernatant of 30 mg of the homogenized cardiac and skeletal tissues by enzyme-linked immunosorbent assay (ELISA), performed according to the manufacturer’s information (PeproTech, Cranbury, NJ, USA). Briefly, 96-well microtiter plates (Nunc) were coated with the TNF, IL-6, IL15, IL-10 and CCL2 monoclonal antibodies (100 μL/well) for 18 h at 4 °C and then washed with PBS buffer (137 mM NaCl, 2.7 mM KCl, 8.1 mM Na_2_HPO_4_ and 1.5 mM KH_2_PO_4_) (pH 7.4) containing 0.05% tween-20. Non-specific binding sites were blocked with 1% bovine serum albumin (200 μL/well) in PBS. Plates were washed and plasma samples were added (100 μL/well) followed by incubation for 18 h at 4 °C. A biotinylated antibody, diluted in blocking buffer containing 0.05% tween-20, was added (100 μL/well) and the plates were incubated further for 1 h at 24 °C. This process was followed by the addition of streptavidin-horseradish peroxidase and, after incubation and washing, chromogenic substrate (o-phenylenediamine (Sigma-Aldrich Brasil Ltda, São Paulo, SP, Brazil) diluted in 0.03 M citrate buffer containing 0.02% of H_2_O_2_) was added (100 μL/well) to incubate in the absence of light at room temperature. The reaction was interrupted by adding 1 M H_2_SO_4_ solution (50 μL/well). The Biotek ELx808 microplate reader (Hayward, CA, USA) was used to measure the absorbance values of the samples, with wavelengths of 450 nm and 630 nm.

Creatine kinase (CK) activity in plasma provides a key marker for cardiac and skeletal muscle disturbances. Blood was collected from infected animals and, plasma isolated by centrifugation at 1000× *g* for 15 min at 4 °C. CK activity was determined using CK-NAC kit (Bioclin biochemical, Belo Horizonte, MG, Brazil) and an Olympus AU640 autoanalyzer (Olympus, Hamburg, German). Briefly, solution 1 (imidazole acetate, glucose, EDTA, NADP, hexokinase, magnesium acetate and N-acetylcysteine) was mixed with solution 2 (glucose-6-phosphate dehydrogenase, creatine phosphate, ADP, AMP and di-adenosine pentaphosphate) in a 4:1 ratio, respectively. The mixture of solution 1 and solution 2 was added to 10 μL of the plasma sample in 1 mL tubes and, after the homogenization, the end-mixture was transferred to a thermoblock heated to a temperature of 38 °C. After 2 min, the content was transferred to a quartz cuvette and placed in the reader a Shimadzu UV-1601 UV-VIS Spectrophotometer with a wavelength of 340 nm. The first reading was performed immediately after the transfer, and two more readings were taken at 2 min intervals. Calibration, quality control of the equipment and data calculation were performed according to the recommended protocol.

Cardiac and skeletal tissue fragments were also used to evaluate histological parameters, such as the area of amastigote nests and the number of infiltrated cells. They were fixed in buffered formalin (10%) for 24 h. They were then dehydrated and fixed in plastic paraffin. After being fixed, the paraffin-embedded tissues were cut (5 μm) and stained with hematoxylin and eosin (HE). The cell nuclei and the area of amastigote nests present in the fragments were quantified in random fields, totalizing 35,463 μm^2^, visualized at 20× magnification. Amastigote nests were highlighted, as inserted, at 40× magnification. A Leica DM5000 B microscope (Leica Application Suite, version 2.4.0R1) and the Leica Qwin V3 image analyzer program were used to digitize and process the acquired images. To count the area of the nests, the Image J program was used.

Data were expressed as means ± standard error of the mean (SEM). To verify the distribution of data, the Kolmogorov–Smirnov test was performed. For parametric data and analysis of variance, the one-way ANOVA test was used, followed by Tukey’s test for multiple comparisons. To evaluate two independent groups, the unpaired t test was used for parametric data, and the Mann–Whitney test for non-parametric data. The GraphPad Prism 8.0.1 software was also used for all analyses. The significance level adopted for the analyzes was *p* ≤ 0.05.

## 3. Results

### 3.1. Theracurmin Has Reduced the Parasitemia Curve and Sustained Mice’s Body Mass

**Figure 1:**
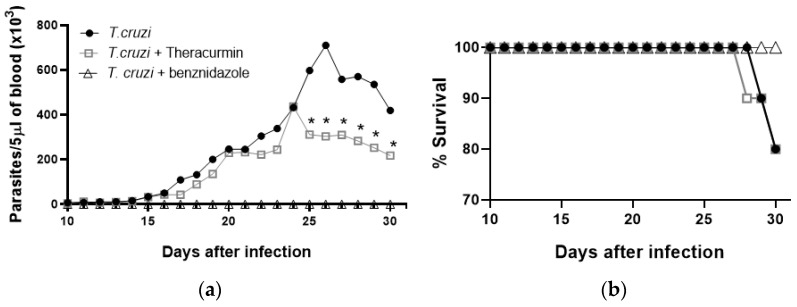
Parasitemia and survival curves in mice infected by *T. cruzi* under Theracurmin or benznidazole therapies: (**a**) number of circulating parasites in Swiss mice treated with Theracurmin during 30 days of infection with the Colombian strain of *T. cruzi* (*n* = 8); (**b**) survival rate of animals in the *T. cruzi*, *T. cruzi* + Theracurmin and *T. cruzi* + benznidazole groups (*n* = 8). * *p* < 0.05 by Mann–Whitney test. Data expressed as mean ± SEM.

Daily use of 30 mg/kg of Theracurmin significantly reduced the number of blood parasites between days 25 and 30 in animals infected with the Colombian strain of *T. cruzi* (Figure 1a), while animals treated with benznidazole presented few blood parasites. Differently from benznidazole therapy, Theracurmin and untreated infected mice promoted similar animals surviving at 30 days post infection (Figure 1b). *T. cruzi* infection reduced the relative body mass variation during the acute phase of the infection, causing a lower body mass gain (Table 1), while benznidazole and Theracurmin were capable of partially ameliorating the body mass gain when compared to the uninfected/untreated and to the Theracurmin-treated animals.

### 3.2. Theracurmin Has Reduced CCL2 and IL-15 Tissue and Increased IL-10 in T. cruzi Infection

**Figure 2:**
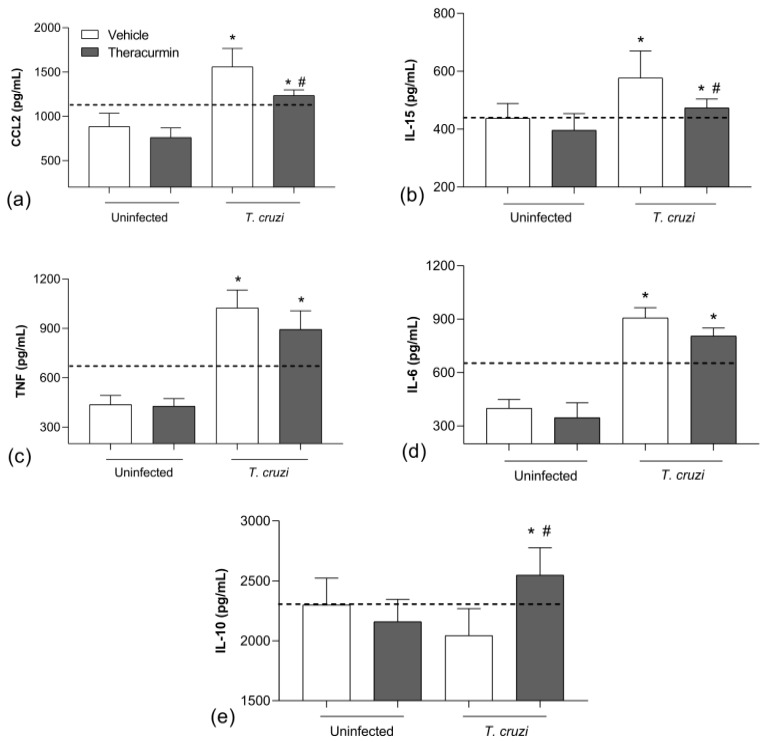
Inflammatory markers in cardiac muscle tissue. The concentration of (**a**) CCL2, (**b**) IL-15, (**c**) TNF, (**d**) IL-6 and (**e**) IL-10 was measured in homogenate of cardiac muscle tissue from those uninfected and *T. cruzi*-infected animals (*n* = 7) under treatment with Theracurmin. Dashed lines mean benznidazole (100 mg/kg) treatment as the positive control. Data expressed as mean ± SEM. * *p* < 0.05 versus uninfected/untreated and Theracurmin groups. # *p* < 0.05 versus *T. cruzi*-infected animals.

**Figure 3:**
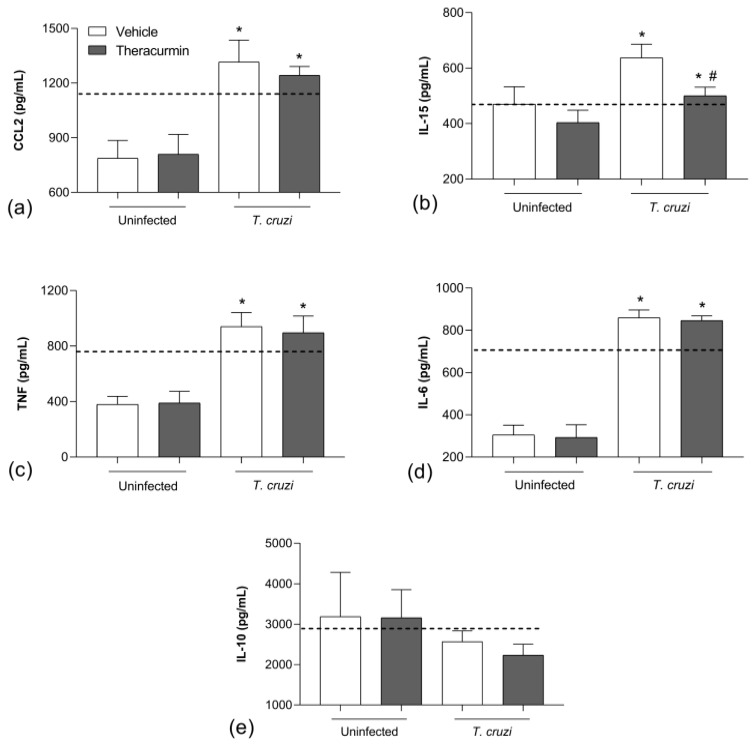
Inflammatory markers in the skeletal muscle tissue. The concentration of (**a**) CCL2, (**b**) IL-15, (**c**) TNF, (**d**) IL-6 and (**e**) IL-10 was measured in homogenate of skeletal muscle tissue from those uninfected and *T. cruzi*-infected animals (*n* = 7) under treatment with Theracurmin. Dashed lines mean benznidazole (100 mg/kg) treatment as the positive control. Data expressed as mean ± SEM. * *p* < 0.05 versus uninfected/untread and Theracurmin groups. # *p* < 0.05 versus *T. cruzi*-infected animals.

*T. cruzi* infection increased the release of TNF, IL-6, IL-15 and CCL2 in cardiac (Figure 2) and skeletal tissues (Figure 3) during the acute phase of experimental infection.

Theracurmin reduced the production of CCL2 (Figure 2a) and IL-15 (Figure 2b) in cardiac tissue, but did not regulate TNF (Figure 2c) and IL-6 (Figure 2d). In addition, the treatment increased the regulatory cytokine IL-10 (Figure 2e) in heart tissue. As for the skeletal tissue, Theracurmin was capable of reducing IL-15 (Figure 3b) but did not regulate the production of CCL2 (Figure 3a), TNF (Figure 3c), IL-6 (Figure 3d) nor IL-10 (Figure 3e). Note that benznidazole therapy was effective in reducing inflammatory mediators and improve the release of IL-10 in the infected animals in both evaluated tissues.

**Figure 4:**
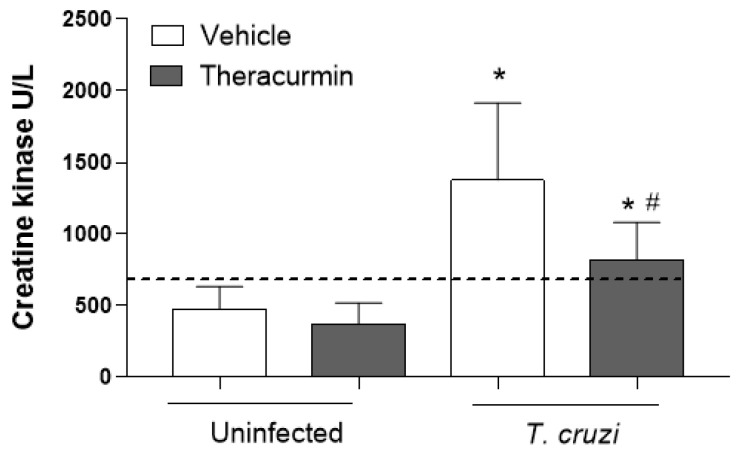
Activity of the plasma creatine kinase during acute *T. cruzi* infection. Creatine kinase (CK) was measured in plasma by biochemical assay in mice infected with Colombian strain of *T. cruzi* at 30 days of infection. Dashed lines mean benznidazole (100 mg/kg) treatment as the positive control. Data are shown as mean of 7 animals ± SEM. * *p* < 0.05 versus uninfected/untreated and Theracurmin groups. # *p* < 0.05 versus *T. cruzi*-infected animals.

The infection elevated the plasma concentration of CK in the animals which suggests muscle damage caused by the parasite. The administration of Theracurmin was able to reduce CK during acute phase of *T. cruzi* infection, in a similar pattern observed in benznidazole therapy (Figure 4).

### 3.3. Theracurmin and Benznidazole Reduced Cardiac and Skeletal Inflammatory Infiltration in Muscles

**Figure 5:**
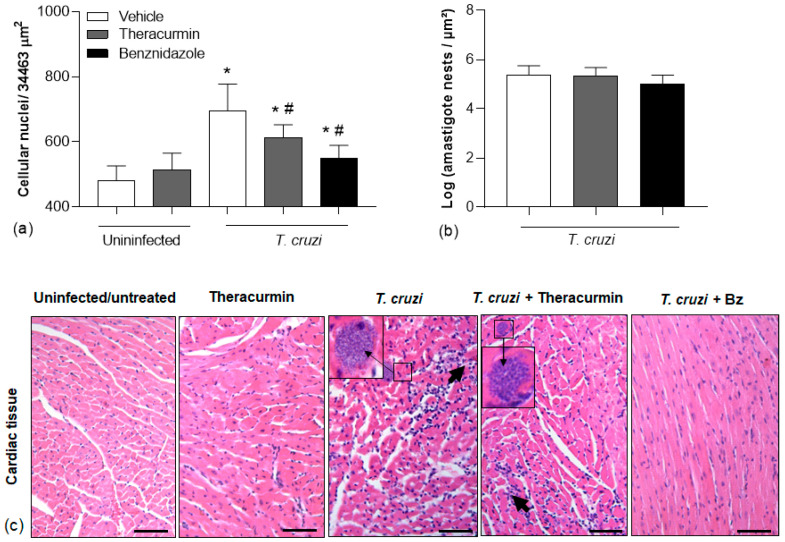
Leukocyte infiltration and amastigote nests in the cardiac muscle tissue. (**a**) The mean cell nuclei in 10 fields were quantified after 30 days of *T. cruzi* infection (Colombian strain); (**b**) indicates the log of *T. cruzi* amastigote forms and (**c**) is a representative image of the histological sections, stained with hematoxylin and eosin, of the cardiac tissue from infected animals treated with Theracurmin or benznidazole. The scale bar corresponds to 50 µm. * *p* < 0.05 uninfected/untreated group versus *T. cruzi +* Theracurmin group. # *p* < 0.05 *T. cruzi* versus *T. cruzi*-infected group.

*T. cruzi* infection induced local or widespread inflammatory foci in infected tissues composed by mononuclear cells (Figure 5). We have observed the increasing inflammatory infiltration in the cardiac tissue from infected animals (Figure 5a). However, Theracurmin and benznidazole seemed to regulate the inflammatory infiltration (Figure 5a) but not the parasitism (Figure 5b,c) in the cardiac tissue.

**Figure 6:**
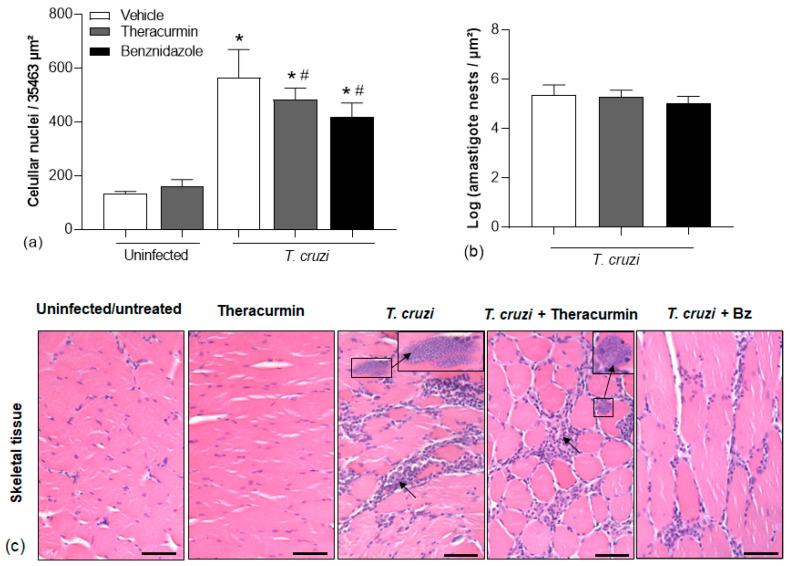
Leukocyte infiltration and amastigote nests in the skeletal muscle tissue. (**a**) The mean cell nuclei in 10 fields were quantified after 30 days of *T. cruzi* infection (Colombian strain); (**b**) indicates the log of *T. cruzi* amastigote forms and (**c**) is a representative image of the histological sections, stained with hematoxylin and eosin, of the skeletal tissue from infected animals treated with Theracurmin or benznidazole. The scale bar corresponds to 50 µm. * *p* < 0.05 versus uninfected/untreated animals and Theracurmin groups. # = *p* < 0.05 versus *T. cruzi*-infected group.

Theracurmin and benznidazole exert similar findings to the skeletal muscle (Figure 6) in terms of leukocyte infiltration (Figure 6a) and the quantification of amastigote forms of the parasite (Figure 6b,c) in the gastrocnemius.

## 4. Discussion

The use of natural products as complementary/alternative elements as treatment for different diseases has been part of human culture for many centuries [19]. This is due to the bioactive characteristics of different substances such as flavonoids, polyphenols and, more recently, curcumin acts against relevant disorders such as metabolic syndrome, neurodegenerative, cancer and cardiovascular [20,21].

In tropical diseases, curcumin has demonstrated useful properties in the controlling of *T. cruzi* and in the inflammatory response concerning this infection. Infected mice that were treated with curcumin have provided a reduction in parasitism, an increase in survival rate, a reduction in cardiac parasitism and they have reduced expression of pro-inflammatory cytokines such as IL-6 and TNF [3,4]. It is still unclear in the literature through which mechanisms curcumin promotes such changes; however, there are indications that its activity is related to changes in the cytoskeleton of the parasite, indicating tubulin as a possible target [22]. In addition, previous studies have also shown that curcumin is capable to inhibit serine/threonine protein kinase activation, which is involved in the constitutive and inducible NF-kB activation pathway [23], as well as inhibit Janus kinase, STAT3 and phosphatases activation. Despite the results, curcumin is a natural compound with low solubility in water at room temperature. Such a fact has made its application complicated in research even though its use is spread in culinary spheres. Thus, the development of Theracurmin was proposed as an attempt to increase solubility and potentialize the effects observed with curcumin [11].

The present study evaluated the oral administration of Theracurmin in experimental infection with the Colombian strain by *T. cruzi*, characterized as being resistant to benznidazole and as promoting a high inflammatory and tissue parasitism [24,25,26]. Our findings suggest a systemic action of Theracurmin on the circulating parasites; however, there seems to be a limitation of this action in the distribution of the natural compound in the tissues. In addition, Theracurmin was capable of reducing the muscle damage and the inflammatory mediators, culminating in a low leukocyte influx into the organs. Furthermore, it is noted that the behavioral pattern of Theracurmin is similar to the bioavailability of benznidazole, acting in the acute phase against *T. cruzi* and with little efficiency in the chronic phase of infection [24]. However, we do not expect its future application to eliminate *T. cruzi* by itself, but as a “complementary” use in association with routine chemical treatments (e.g., benznidazole, nifurtimox or other potential new compounds). It sounds positive since tissue damage is particularly caused by host immune response against parasite antigens, and this supplementary diet could support act minimizing this immunopathological condition. In this sense, our group has recently shown (unpublished data) an immune action of Theracurmin in a specific concentration–dose–response curve. Studies have reported the association of curcumin with benznidazole by, during 20 days of infection, applying the Y strain of the parasite in which there was a reduction of the cardiac inflammation and in the oxidative damage in addition to attenuating the liver toxicity induced by benznidazole [27]. The reduction in the parasitemia curve in the face of a benznidazole-resistant strain signals a promising future for further studies on the additive effect of the curcumin/Thercurmin or linked to new possibilities as to the distribution of these natural and chemical compounds using nanocapsules [28], including being investigated in different models of *T. cruzi* infection.

One of the most relevant findings in *T. cruzi* infection in experimental models and humans is the progressive tissue inflammatory process, which occurs with the aim of eliminating trypomastigote and amastigote forms of the parasite but culminates in a tissue destruction and declined functions of the organs. In the context of the cellular immune response, the Colombian strain of *T. cruzi* increases the production of pro-inflammatory mediators, such as IFN-γ, TNF, IL-6, IL-12, CCL2, CCL3, CCL5, CXCL10 and CX3CL1, among others [14,29,30]. The interaction between innate and acquired immune responses to control *T. cruzi* during early stage of infection is critical to the host surviving. Part of this parasite-controlling response involves neutrophils, macrophages, natural killer cells, T and B cells that can be activated by activation of primitive receptors (e.g., Toll-like receptors 2, 4, 7 and 9 and nucleotide oligomerization domain-like receptors 1 and NLRP3 inflammasome), by antigen-presenting cells or by NK-kB activation as promoters of a set of systemic and local inflammatory mediators [31,32,33,34]. However, *T. cruzi* has adapted to synthesize specialized pro-resolving mediators of inflammation (thromboxane A2, eicosanoids, resolvins) and angiogenic factors (vascular endothelial growth factor, angiopoietin-1 and 2), which could modulate the parasite microenvironment, allowing its surviving [35,36,37]. Therefore, the ideal treatment for *T. cruzi* should be capable of eliminating parasites with minimum or no toxicity and capable of mitigating the tissue damage in distinct organs caused by an exacerbated inflammatory response against parasite molecules. In the context of *T. cruzi* infection, our group has investigated the potential parasitological and immunomodulatory effects of drugs (e.g., enalapril maleate, simvastatin, beta-blocker, doxycycline) and naturally derived compounds (e.g., xanthenodiones and tetraketones) in a single therapy and/or in combination with Bz [9,24,38,39]. Supported by the promising data reflecting partial elimination of *T. cruzi* and modulation of the immune response (systemically and in infected tissues), our group has moved forward investigating curcumin and/or the submicron dispersed formulation of the curcumin, Theracurmin, in *T. cruzi*-infected mice.

Our study has shown that the inflammatory mediators TNF, IL-6, IL-15 and CCL2 were elevated in the presence of the parasite, both in cardiac muscle tissue (heart) and in skeletal muscle tissue (gastrocnemius). Thus, Theracurmin has reduced the production of CCL2 and IL-15 in the evaluated tissues, a fact that suggests a reduction in the recruitment of mononuclear cells to the inflammatory site in late stages of infection. In addition, Theracurmin therapy has promoted an elevation of tissue IL-10, which suggests a modulation of a local tissue inflammation and, in perspective, a better myocarditis prognosis in the animals [40]. CCL2 has been identified as an essential chemokine for the generation of the inflammatory pattern associated with the Colombian and other strains of *T. cruzi*, it has been investigated in different times of experimental infection [14,24,25]. The high CCL2 production is coincident with the presence of parasites and mediates mononuclear cells recruitment into tissues and, in chronic stage of the human disease, this chemokine was suggested as a potential marker of cardiac dysfunction [14]. This regulatory role of Theracurmin on CCL2 and IL-15 is partially explained by in vitro and in vivo experiments demonstrating a down-regulation on the mitogen-activated protein kinase and NF-kB signaling [41,42], essential keys to regulate gene expression of proinflammatory mediators.

The IL-15 has presented itself as a multifunctional cytokine that plays an important role in the proliferation and development of T and NK cells, in addition to modulating the host response against intracellular pathogens [43]. This cytokine seems to be involved in tissue damage in patients with chronic Chagas cardiomyopathy due to its role in maintaining CD8+ T cells [44]. Therefore, IL-15 and CCL2 are induced by tumor necrosis factor, a key cytokine responsible for the magnificence of the acute and chronic immune responses in mice [45] and human [46] infections by *T. cruzi*. This inflammatory mediator network promotes activation and leukocyte chemotaxis from the blood into the infected tissues to eliminate parasites. We demonstrated that in a systemic way, Theracurmin was responsible for reducing IL-15; however, due to the acute period of infection, it was not possible to visualize this regulatory pattern applied in chronic cardiac and skeletal muscle protection. 

Finally, the use of benznidazole therapy as a positive control in this study reinforced its property of reducing circulating parasites (Colombian strain), with a minor impact on tissue parasitism and its immunomodulatory properties on plasma and tissue-derived mediators during *T. cruzi* infection, as previously shown by our group [17,24,37,38]. Indeed, results regarding benznidazole were better and/or similar to those observed to Theracurmin. However, the high toxicity caused by benznidazole is still a reality and has a negative impact on the treatment of Chagas disease, which instigates investigations on new complementary therapies using natural compounds and anti-parasitic nutraceutical agents.

In conclusion, this study highlights findings underlying the oral administration of Theracurmin and the benefits in *T. cruzi* control and in the inflammatory response caused by the Colombian strain of *T. cruzi* in experimental infection. In the last few years, Theracurmin has been tested in humans, showing a better absorption efficiency then other ordinary curcumin beverages and, in phase I of the study for cancer treatment, Theracurmin presented good tolerance and no side effects during 9 months of oral intake, even when associated with other cancer therapies [47,48]. These encouraging studies open up the potential for future investigations of Theracurmin analogues, alone and in combination with benznidazole and other potential antiparasitic agents.

## Figures and Tables

**Table 1:** Body mass variation. Body mass variation (final body mass − initial body mass/initial body mass × 100) of Swiss mice infected with the Colombian strain and treated with Theracurmin and benznidazole for 30 days. Data expressed as mean ± SEM.

Uninfected and Untreated (%)	Theracurmin (%)	*T. cruzi* (%)	*T.cruzi* + Theracurmin (%)	*T. cruzi +* Benznidazole (%)
12.94 ± 0.96	9.718 ± 1.5	0.7850 ± 2.5 *	1.995 ± 3.4 *^,#^	3.072 ± 4.2 *^,#^

* *p* < 0.05 when compared to the uninfected/untreated animals. ^#^ *p* < 0.05 when compared to the *T. cruzi*-infected group.

## Data Availability

The data presented in this study are available on request from the corresponding author.
